# A panel of eight mRNA signatures improves prognosis prediction of osteosarcoma patients

**DOI:** 10.1097/MD.0000000000024118

**Published:** 2021-04-09

**Authors:** Bo Wu, Zhan Wang, Nong Lin, Xiaobo Yan, Zhangchun Lv, Zhimin Ying, Zhaoming Ye

**Affiliations:** a Department of Orthopaedics, YongKangShi Hospital of Traditional Chinese Medicine, Yongkang; b Department of Orthopaedics, The Second Affiliated Hospital of Zhejiang University School of Medicine, Hangzhou, P.R. China.

**Keywords:** genes, osteosarcoma, prognosis, signature, therapeutically applicable research to generate effective treatments database

## Abstract

Genetic alterations are vital to the progression of osteosarcoma carcinoma. The present study investigated a panel of gene signatures that could evaluate prognosis in osteosarcoma based on data from the Therapeutically Applicable Research To Generate Effective Treatments initiative. Osteosarcoma messenger RNA (mRNA) profiles and clinical data were downloaded from the therapeutically applicable research to generate effective treatments database. Patients with osteosarcoma were divided into two groups based on findings at diagnosis: with and without metastasis. Differentially expressed mRNAs were compared and analyzed between groups. Univariate and multivariate Cox regression analyses identified a set of eight mRNAs with the ability to classify patients into high-risk and low-risk groups with significantly different overall survival times. Further analysis indicated that the eight-mRNA signature was an independent prognostic factor after adjusting for other clinical factors. Receiver operating characteristic curve analysis demonstrated a good performance of the eight-mRNA signature. Further, the biological processes and signaling pathways of the eight-mRNA signature were reviewed using Gene Ontology and Kyoto Encyclopedia of Genes and Genomes resources. Finally, the results of the TCGA analysis were verified by other cohorts from Gene Expression Omnibus database. The identification of an eight-mRNA signature not only provides a prognostic biomarker of osteosarcoma but also offers the potential of novel therapeutic targets for its treatment.

## 1 Introduction

Osteosarcoma is the most common type of primary malignant bone cancer, constituting 15% of all diagnosed bone tumor cases.^[1–4]^ It is a highly heterogeneous malignancy, with a large percentage of patients having a distant metastasis when first diagnosed.^[5,6]^ The current standard treatment^[7,8]^ is a combination of local control surgery with post/preoperative systemic multi-agent chemotherapy including cisplatin, epirubicin, etopside, methotrexate, and cyclophosphamide.^[9,10]^ Although the standard therapy has been proven to be an effective treatment for osteosarcoma, patients still have a poor prognosis with a greater than 40% 5-year mortality rate.^[11–13]^ Therefore, it is important to increase our understanding of the underlying molecular mechanisms of osteosarcoma, and to establish a molecular biomarker model for diagnosis and prognosis to improve patient outcome.

With the rise of genetic sequencing, we now have a better understanding of messenger RNAs (mRNAs). These are a large family of RNA molecules that convey genetic information from DNA to the ribosome, where they specify the amino acid sequence of the protein products of gene expression. A large number of differentially expressed mRNAs have been observed in tumors compared with healthy adjacent tissues. mRNAs are closely associated with cell differentiation, proliferation, and apoptosis, and accumulating evidence indicates that dysregulated mRNAs play an oncogenic or tumor suppressor role in cancer development.^[14–17]^ Given the fundamental roles and intrinsic characteristics of mRNAs, many have been regarded as diagnostic and prognostic biomarkers in cancers, including BRAF,^[18]^ KRAS,^[19]^ and Her-2.^[20]^ Although several studies have previously classified mRNA biomarkers for prognosis prediction in osteosarcoma,^[21–23]^ a systematic investigation of the prognostic significance of mRNA signatures in osteosarcoma patients has not been undertaken.

The present study downloaded and analyzed mRNA expression profiles of a large number of osteosarcoma patients from the Therapeutically Applicable Research To Generate Effective Treatments (TARGET) project to systematically investigate the prognostic significance of mRNAs. We identified eight biologically relevant mRNAs that were significantly connected with the prognosis of osteosarcoma patients using survival analysis and Cox regression analysis. These were used to establish an eight-mRNA signature that was confirmed as an independent prognostic factor for use in predicting overall survival in patients with osteosarcoma.

## 2 Materials and methods

### 2.1 Data source

RNA sequencing (RNA-seq) data and clinical information about osteosarcoma patients in the TARGET project were downloaded from the Genomic Data Commons Data Portal (https://portal.gdc.cancer.gov/) in October 2018. First, RNA-seq data files were merged into a matrix file using the merge script of the Perl language (http://www.perl.org/). Then, the gene name was converted from the Ensembl id to the matrix of the gene symbol through the Ensembl database (http://asia.ensembl.org/index.html). We selected mRNA data derived from osteosarcoma tumor tissue samples. A total of 101 osteosarcoma TARGET mRNA data samples were downloaded from the database. According to the findings at diagnosis, patients were divided into two groups: Group NM (osteosarcoma patients without metastasis) and Group M (osteosarcoma patients with metastasis). The R package edgeR was used to identify genes that were differentially expressed (DEGs) between Group NM and Group M, with false discovery rate <0.05 and |logFC|>2 set as the threshold. Gene expression levels based on microarray data were calculated using R package limma with RMA correction.

### 2.2 Gene information acquisition and clinicopathological features

Gene information was obtained from downloaded data. We excluded patients with incomplete clinicopathological parameters and those with missing prognostic follow-up data. A total of 95 patients were analyzed in our study.

### 2.3 mRNA-related prognostic model

To discover potential mRNA factors affecting the prognosis of osteosarcoma patients, univariate Cox proportional hazard regression analysis was applied using the R survival package to evaluate the association between mRNA expression and overall survival. Only those mRNAs with *P* < .05 were considered candidate mRNA prognostic markers of osteosarcoma. Multivariate Cox proportional hazards regression was performed to identify optimal prognostic mRNAs that impact on the survival of osteosarcoma patients. An individual's risk score model was established to evaluate survival risk as follows:


Risk Score (RS)=Σcoefficient (mRNAi)×expression (mRNAi)

Here, mRNAi is the identifier of the ith selected mRNA. The risk score model was a measure of prognostic risk for each osteosarcoma patient.

### 2.4 Risk stratification and ROC curve

The risk score was calculated according to the predictive mRNA signature model. Using the median risk score as the cutoff, enrolled patients were classified into high-risk and low-risk groups. A high-risk score indicates poor survival for osteosarcoma patients. Kaplan-Meier analysis was used to generate overall survival curves, and log-rank tests were performed to assess overall survival differences between high-risk and low-risk patient groups. The performance of the mRNA prognostic model was evaluated by calculating the area under the curve (AUC) of the receiver operating characteristic (ROC) curve in the R package of “survival ROC”. Univariate and multivariate analyses with Cox proportional hazards regression for overall survival were performed on individual clinical risk factors with and without the eight-mRNA signature. Hazard ratios and 95% confidence intervals were estimated.

### 2.5 Functional enrichment analysis

The mRNA data samples from osteosarcoma tumor tissues in the TARGET project were downloaded from the Genomic Data Commons Data Portal. According to univariate and multivariate analyses, eight prognostic mRNAs were associated with the prognosis of osteosarcoma patients. Pearson correlation coefficients between the expression profiles of the eight prognostic mRNAs and co-expressed protein-coding genes (PCGs) were computed to identify co-expression relationships of mRNAs and PCGs. PCGs with |Pearson correlation coefficient| >0.40 and *P* < .01 were considered to be mRNA-related PCGs. Functional enrichment analysis of PCGs with eight prognostic mRNAs was conducted for gene ontology (GO) and Kyoto Encyclopedia of Genes and Genomes (KEGG) pathways using Database for Annotation, Visualization and Integrated Discovery Bioinformatics Resources (version 6.8). Significant functional categories were identified when adjusted *P* values by Benjamini were <.1.

### 2.6 GEO database verification

The datasets of osteosarcoma patients were obtained from the Gene Expression Omnibus (GEO) database (http://www.ncbi.nlm.nih.gov/geo/). GSE39058 was selected in the present study and the expression profiles were normalized by log2-conversion, the clinical characteristics of which are also attached.

### 2.7 Statistical analysis

Statistical evaluation was performed with SPSS 21.0 software package (IBM, Armonk, NY). Measurement data are presented as means ± standard deviations (X ± S). Continuous variables with normal distributions were tested by independent sample t-tests between intergroup comparisons; otherwise, the one way Mann-Whitney test was used. Enumeration data are presented as frequency and rate. Chi-square tests were used to analyze categorical variables. Univariate and multivariate Cox regression analyses were used to identify factors that were related independently to the outcome of osteosarcoma patients. Prognostic performance was estimated by ROC analysis. *P* < .05 was considered statistically significant.

## 3 Results

### 3.1 Clinical data of samples

Ninety-five patients were analyzed in our study. Their clinical data are shown in Table 1.

**Table 1:** Clinical data of the 95 patients.

Covariates	Number of patients (N = 95)	Proportion of patients (N% = 100%)
Age		
≤14	42	44.2%
>14	53	55.8%
Sex		
Male	55	57.9%
Female	40	42.1%
Ethnicity		
White	74	77.9%
Asian	9	9.5%
African	12	12.6%
Relapse or metastasis		
Yes	46	48.4%
No	49	51.6%
Primary tumor site		
Around the knee joint	74	77.9%
Beyond the knee	21	22.1%
Tumor necrosis rate		
≤90	55	57.9%
>90	40	42.1%

### 3.2 Differentially expressed mRNAs between Group NM and Group NM osteosarcoma patients

**Figure 1:**
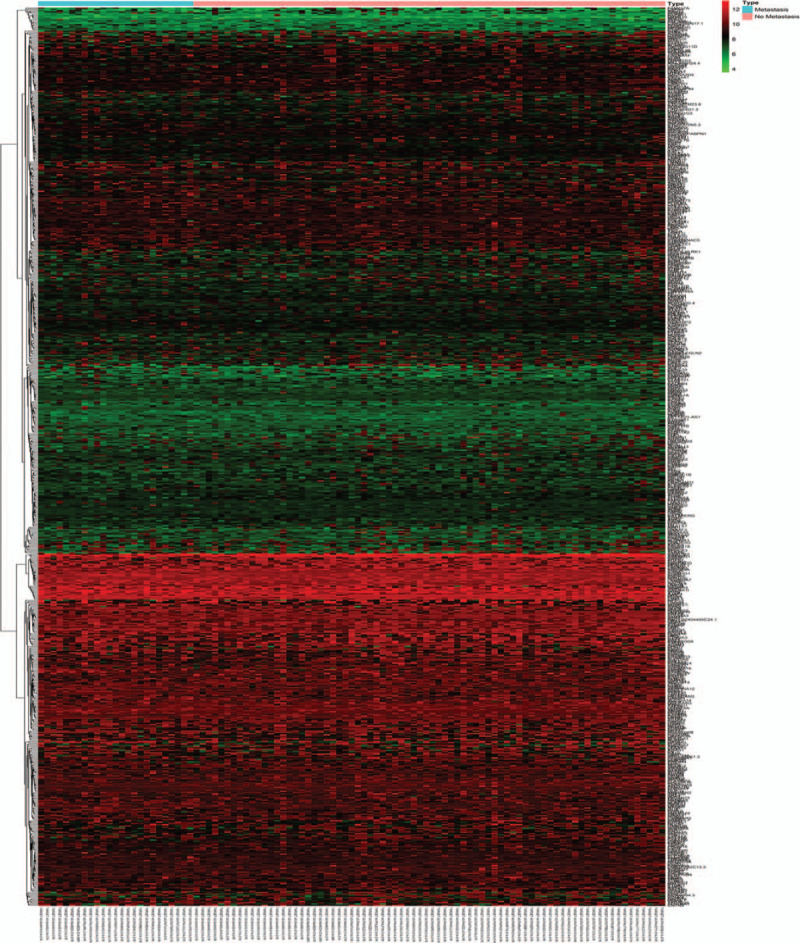
mRNA expression in Group NM and Group M patients from the TARGET database. TARGET: therapeutically applicable research to generate effective treatments.

mRNA expression in Group NM and Group M was investigated using gene expression information from TARGET database profiles. In total, 701 differentially expressed mRNAs were identified between Group NM and Group M patients, of which 404 were upregulated and 297 downregulated (Fig. 1).

### 3.3 Identification of prognostic mRNAs

To identify prognostic mRNAs, expression data of each differentially expressed mRNA were subjected to univariate Cox proportional hazards regression analysis. This identified 14 mRNAs that were significantly associated with overall survival (adjusted *P* < .01). Multivariate Cox proportional hazards regression was used to select the optimal eight mRNAs as independent remarkable prognostic factors (Table 2).

**Table 2:** Eight mRNAs selected as prognosis-associated factors in osteosarcoma.

mRNA	*P* value^∗^	Hazard ratioa^∗^	Coefficienta^∗^
UGT3A2	.0055	2.1442	0.7661
SAXO2	.0015	0.3788	–1.0487
PLEKHG1	.0002	0.2536	-0.8840
DSCR8	.0021	1.5767	0.6846
MYL1	.0061	1.7141	0.6977
TMEM125	.0003	0.2926	-0.8993
GAGE1	.0082	1.3112	0.3215
SCN1A	.0078	0.5117	-1.1019

∗ Derived from the multivariate Cox regression analysis.

Among these eight prognostic mRNAs, four were risk factors because they had positive coefficients in Cox regression analysis and their high expression was associated with a shorter overall survival time. The other four mRNAs could be regarded as protective factors because their high expression was closely associated with longer patient survival.

### 3.4 The eight-mRNA prognostic model

**Figure 2:**
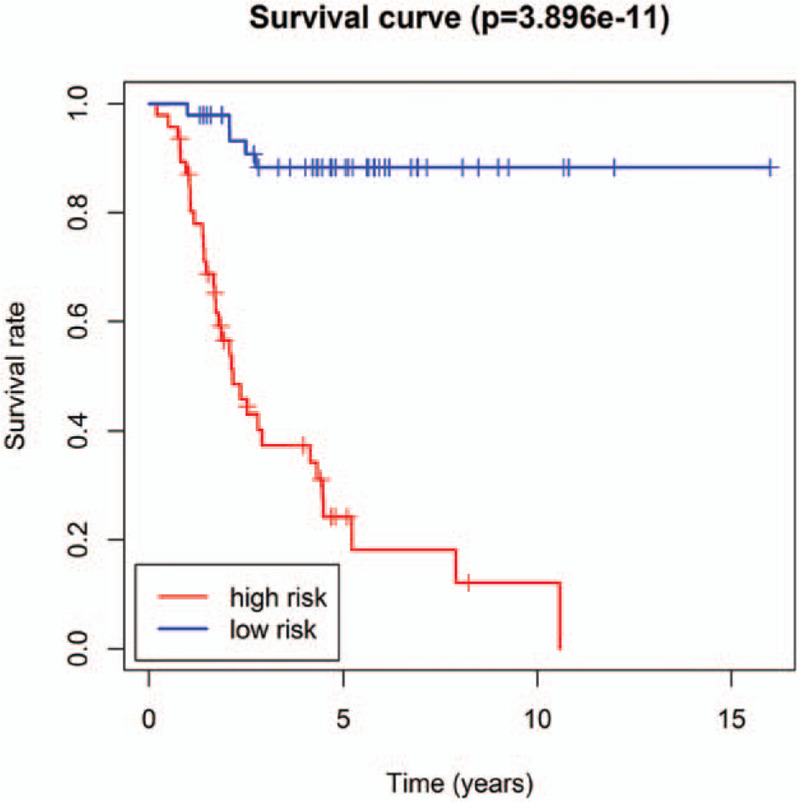
Kaplan-Meier curve analysis of overall survival in high-risk and low-risk osteosarcoma patients.

The eight prognostic mRNAs were combined to establish the eight-mRNA signature, which is associated with the overall survival of osteosarcoma patients. This signature was developed as a linear combination of the expression levels of the eight mRNAs weighted by their relative regression coefficients in multivariate Cox regression as follows: Risk Score = (0.7661 × expression value of UGT3A2) + (-1.0487 × expression value of SAXO2) + (-0.8840 × expression value of PLEKHG1) + (0.6846 × expression value of DSCR8) + (0.6977 × expression value of MYL1) + (-0.8993 × expression value of TMEM125)+ (0.3215 × expression value of GAGE1)+ (-1.1019 × expression value of SCN1A). According to the risk score model, we calculated an mRNA expression-based risk score for each patient and classified them into high-risk (n =47) or low-risk groups (n = 48) using the median risk score as the cutoff point. The overall survival of the two groups differed significantly (*P* = 3.896e−11, log-rank test) (Fig. 2). The median survival time for patients in the high-risk and low-risk groups was 1.8 and 4.9 years, respectively, while overall survival at 5 years was 18.1% and 88.3%, respectively. The univariate Cox regression model of overall survival indicated that the high-risk group was more likely to have higher mortality than the low-risk group (odds ratio 12.66 [95% confidence interval 4.891–32.750, *P *< .0001]) (Table 3).

**Table 3:** Univariate and multivariate Cox regression analysis of overall survival in TARGET osteosarcoma data set.

		Univariate analysis			Multivariate analysis		
Variables		HR	95% CI of HR	*P* value	HR	95% CI of HR	*P* value
Age	≤14/>14	1.290	0.682–2.441	.433	1.438	0.720–2.986	.741
Sex	male/female	0.972	0.506–1.866	.931	0.854	0.421–1.706	.539
Ethnicity	white/asian/african	0.893	0.329–2.425	.825	0.702	0.218–2.269	.403
Relapse or metastasis	Yes/No	17.451	5.343–56.991	.000	16.032	4.751–51.208	.000
Primary tumor site	Around the knee joint/beyond the knee	0.862	0.611–1.325	.511	1.025	0.733–1.648	.759
Tumor necrosis rate	≤90/>90	5.318	1.215–23.284	.007	4.372	1.017–21.853	.026
Eight-mRNA risk model	high/low	12.656	4.891–32.750	.000	10.126	4.037–29.865	.000

CI = confidence interval, HR = hazard ratio, TARGET = therapeutically applicable research to generate effective treatments.

### 3.5 ROC curve analysis and risk stratification of the eight-mRNA signature

**Figure 3:**
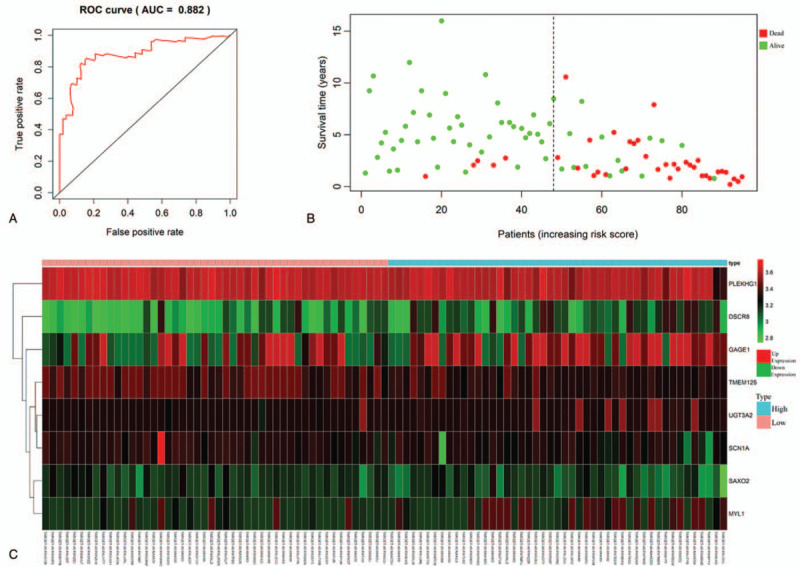
The eight-mRNA signature significantly associated with survival in patients with osteosarcoma. A: Time-dependent ROC curve analysis of the eight-mRNA signature. B: Survival status for patients in high-risk and low-risk groups according to the eight-mRNA signature. C: mRNA expression for patients in high-risk and low-risk groups according to the eight-mRNA signature. ROC: receiver operating characteristic.

Time-dependent ROC curves were used to measure the predictive performance of the eight-mRNA prognostic risk model. The optimal risk score cutoff obtained from ROC curve analysis was 0.730 (sensitivity 86.8% and specificity 75.4%). The AUC of the ROC curve for the eight-mRNA prognostic model was 0.882 at 5 years overall survival (Fig. 3A). The survival status of the osteosarcoma patients is shown on the dot plot (Fig. 3B), and the heatmap shows expression patterns of the eight prognostic mRNAs between high-risk and low-risk groups (Fig. 3C). In the high-risk group, the four risk mRNAs were up-regulated and the four protective mRNAs were down-regulated compared with the low-risk group. The high expression of protective mRNAs in the low-risk group was associated with longer survival time.

### 3.6 The eight-mRNA signature prognostic value is independent of other clinical variables

To evaluate whether the prognostic value of the eight-mRNA signature was independent of other clinical variables, we performed multivariate Cox regression analysis using age, sex, ethnicity, relapse or metastasis, primary tumor site, tumor necrosis rate, and prognostic risk score model as co-variables. We observed that the eight-mRNA signature maintained an independent correlation with overall survival when adjusted for age, sex, ethnicity, relapse or metastasis, primary tumor site, and tumor necrosis rate (Table 3). However, relapse or metastasis and the tumor necrosis rate were significant in the multivariate analysis. Therefore, stratification analysis was performed to examine the independence of the eight-mRNA signature according to relapse or metastasis and tumor necrosis rate.

**Figure 4:**
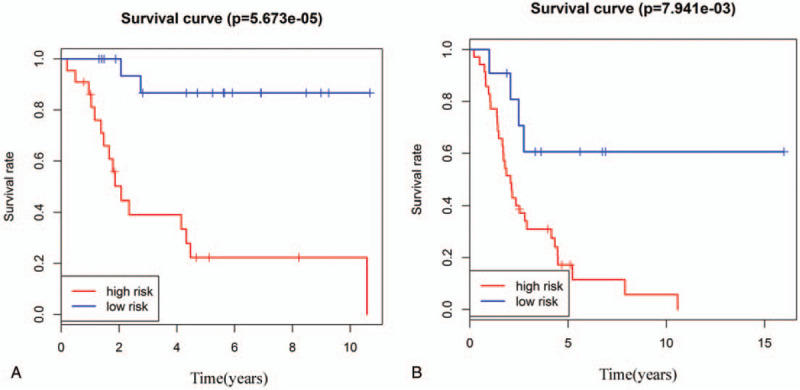
Stratification analysis by relapse or metastasis. A: Kaplan-Meier curve analysis of overall survival in high-risk and low-risk groups for patients without relapse or metastasis. B: Kaplan-Meier curve analysis of overall survival in high-risk and low-risk groups for patients with relapse and metastasis.

**Figure 5:**
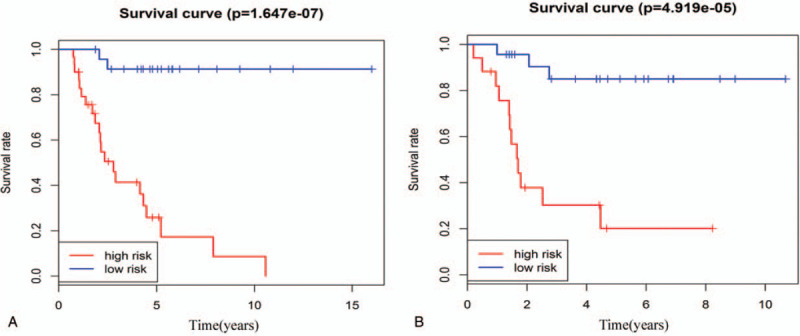
Stratification analysis by tumor necrosis rate. A: Kaplan-Meier curve analysis of overall survival in high-risk and low-risk groups for patients with tumor necrosis rate ≤90%. B: Kaplan-Meier curve analysis of overall survival in high-risk and low-risk groups for patients with tumor necrosis rate >90%.

All 95 osteosarcoma patients were stratified into two groups either with relapse and metastasis (n = 46) or without relapse or metastasis (n = 49). Then, according to the eight-mRNA signature, patients without relapse or metastasis were classified into high-risk or low-risk groups. We detected a significant difference in overall survival between high-risk and low-risk groups (log-rank test *P* = 5.673e−05) (Fig. 4A). Similarly, patients with relapse and metastasis were divided into the high-risk group with shorter overall survival or the low-risk group with longer overall survival (log-rank test *P* = 7.941e−03) (Fig. 4B). Osteosarcoma patients were also stratified into those with a tumor necrosis rate ≤90% (n = 55) and those with a tumor necrosis rate >90% (n = 40). We found that the eight-mRNA signature separated patients in each of these groups into high-risk or low-risk groups, with associated significant differences in overall survival (log-rank test P = 1.647e−07 for the tumor necrosis rate ≤90% patient group and log-rank test pP = 4.919e−05 for the tumor necrosis rate >90% patient group) (Fig. 5A, B).

### 3.7 Functional implication of the eight-mRNA signature

**Figure 6:**
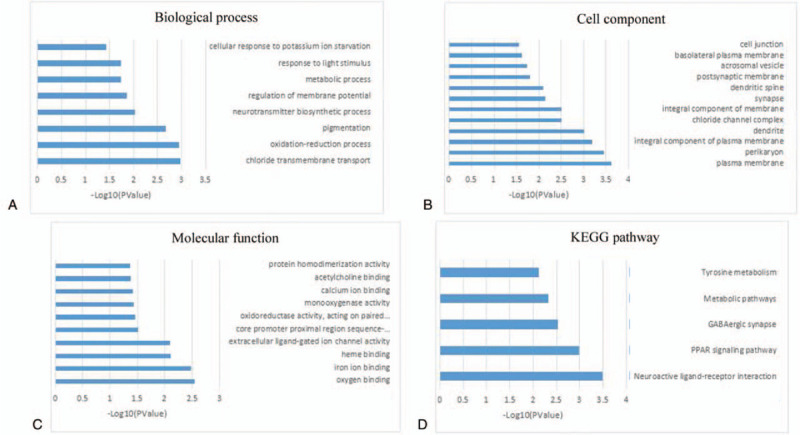
Functional enrichment analysis of protein-coding genes co-expressed with prognostic mRNAs. A: Significantly enriched GO Biological Process terms of eight-mRNAs correlated with protein-coding genes. B: Significantly enriched GO Cell Component terms of eight-mRNAs correlated with protein-coding genes. C: Significantly enriched GO Molecular Function terms of eight-mRNAs correlated with protein-coding genes. D: Significantly enriched KEGG pathways of eight-mRNAs correlated with protein-coding genes. GO = gene ontology; KEGG = Kyoto encyclopedia of genes and genomes.

To explore the functional implication of the eight-mRNA signature, we performed GO and KEGG functional enrichment analysis to reveal particular functional categories of co-expressed PCGs. We measured the co-expression relationships of mRNAs and PCGs by calculating Pearson's correlation coefficient between the expression profiles of the eight prognostic mRNAs and PCGs. PCGs with |Pearson correlation coefficient| >0.40 and *P* < .01 were considered to be mRNA-related PCGs. Functional enrichment analysis demonstrated that co-expressed PCGs clustered most significantly in eight GO Biological Process (BP) terms (Fig. 6A), 12 GO Cell Component (CC) terms (Fig. 6B), 10 GO Molecular Function (MF) terms (Fig. 6C), and five KEGG pathways (Fig. 6D). For GO BP terms, co-expressed PCGs were mainly enriched in chloride transmembrane transport, the oxidation-reduction process, and pigmentation. For GO CC terms, co-expressed PCGs were mainly enriched in the plasma membrane, the perikaryon, and dendrites. For GO MF terms, co-expressed PCGs were mainly enriched in oxygen binding, iron ion binding, and heme binding. Five KEGG pathways were enriched, which mainly focused on neuroactive ligand-receptor interactions, the peroxisome proliferator-activated receptor (PPAR) signaling pathway, GABAergic synapses, and metabolic pathways.

### 3.8 GEO verification

**Figure 7:**
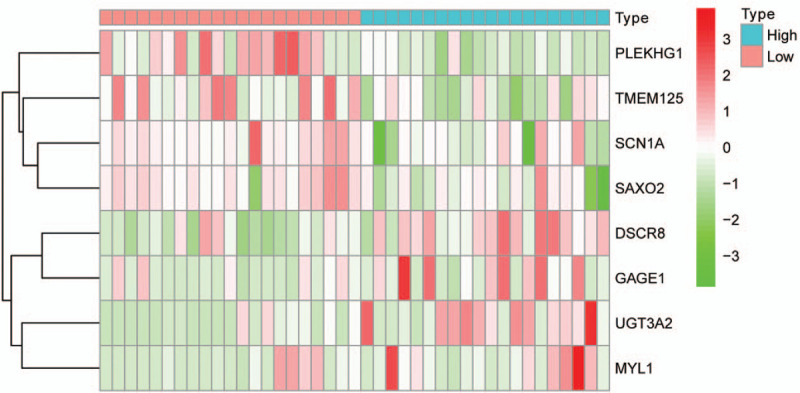
The expression of eight mRNA between high-risk and low-risk groups in GSE39058 cohort.

**Figure 8:**
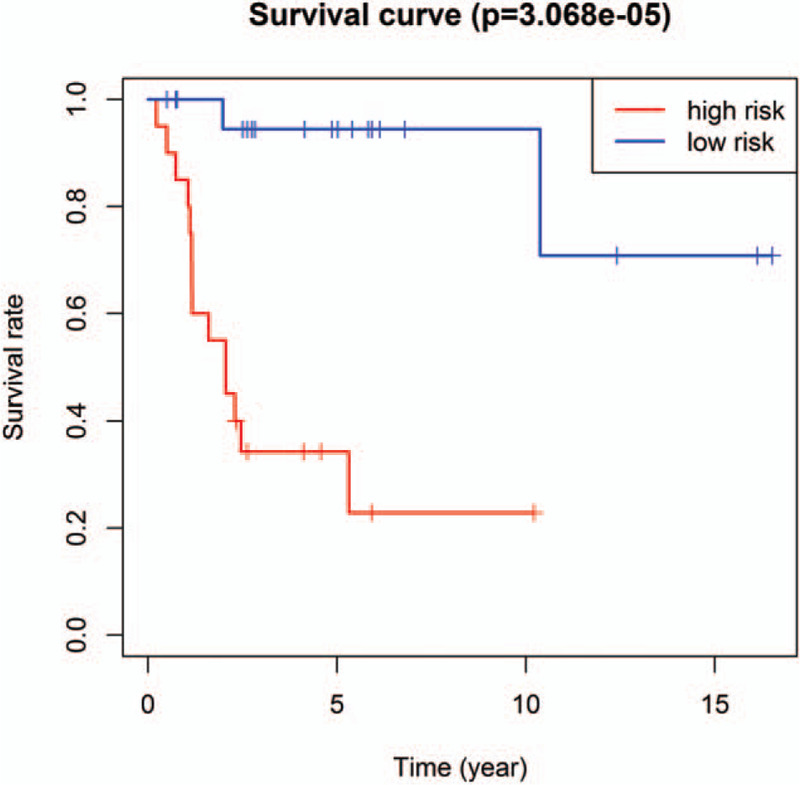
Kaplan–Meier curve analysis of overall survival in high-risk and low-risk osteosarcoma patients in GSE39058 cohort.

In order to further confirm the previous findings from TCGA analysis, we selected GSE39058 to verify the accuracy of the above results. According to the risk score model, we calculated an mRNA expression-based risk score for each patient and classified them into high-risk (n = 20) or low-risk groups (n = 21) using the median risk score as the cutoff point. The expression of eight selected mRNA were significantly different between high- and low-risk groups (*P* < .05) (Fig. 7). Moreover, the overall survival of the two groups was significantly different; patients in the high-risk group had a shorter overall survival compared with patients in low-risk group (Fig. 8; *P* = 3.068e−05). The outcomes of GEO datasets were consistent with results of the abovementioned TCGA profiles.

## 4 Discussion

Although current standard treatments have improved the survival of osteosarcoma patients, their outcome remains unsatisfactory. During the past decade, several conventional prognostic systems for osteosarcoma have been proposed to predict patient prognosis. These systems typically take into account a series of clinical characteristics, such as age, sex, surgical approach, tumor necrosis rate, and chemotherapy, but often result in an insufficient prediction for risk classification and clinical outcome evaluation. Recent advances have demonstrated the molecular heterogeneity of osteosarcoma, which underlies the fact that individual patients have different prognoses and tumor responses to therapy.^[24,25]^ Thus, novel molecular prognostic indicators that can effectively predict the prognosis of osteosarcoma require identification. Given their inherent characteristics and importance, accumulating evidence has revealed roles for mRNAs in cancer diagnosis and prognosis.^[26–32]^

In this study, we investigated the prognostic value of mRNAs by analyzing the correlation between mRNA expression and overall survival in osteosarcoma patients. Using Cox regression analysis and risk scoring methods, we identified eight mRNAs that were significantly associated with the outcome of osteosarcoma patients. We developed an eight-mRNA signature that categorized osteosarcoma patients into high-risk and low-risk groups with significantly different overall survival times. ROC analysis achieved an AUC of 0.882, demonstrating the high sensitivity and specificity of the eight-mRNA signature in predicting prognosis for osteosarcoma patients.

Multivariate Cox analysis also revealed that the eight-mRNA signature is independent of conventional clinical factors, including age, sex, ethnicity, relapse or metastasis, primary tumor site, and tumor necrosis rate. Additionally, we found the eight-mRNA signature clearly distinguished patients at high-risk from those at low-risk within subgroups by performing subgroup stratified analysis. Taken together, these results indicate that the eight-mRNA signature has the potential to allow clinicians to determine and select individualized and effective treatment for patients with different clinical or molecular characteristics.

Recently, a wealth of evidence has revealed that mRNAs show over-expression or reduced expression in the development of osteosarcoma.^[33–35]^ Furthermore, many studies demonstrate that mRNAs are involved in a diverse range of biological processes by regulating protein translation,^[36,37]^ and that co-expressed genes are often involved in the same process or signaling pathway.^[38]^ Therefore, it is reasonable to reveal potential roles of the eight-mRNA signature by functional views of PCGs that are co-expressed with the prognostic mRNAs. We found that the prognostic mRNAs were enriched in chloride transmembrane transport, the oxidation-reduction process, pigmentation, the plasma membrane, perikaryon and dendrites, oxygen binding, iron ion binding, heme binding, neuroactive ligand-receptor interaction, the PPAR signaling pathway, GABAergic synapses, and metabolic pathways, which were closely associated with cellular metabolism, cell activity, and tumor regression. Neuroactive ligand-receptor interactions are considered to be critical in apoptosis, cell proliferation, and metastasis.^[39,40]^ Apoptosis and cell proliferation are associated with neoplasia and tumor progression. PPARs (Peroxisome proliferator-activated receptors) are ligand-activated transcription factors that are crucial regulators of glucose and lipid metabolism, cellular homeostasis, and inflammation.^[41,42]^ Moreover, cellular growth and differentiation are key processes for carcinoma development and progression. The findings in our study are consistent with recently reported results,^[43]^ which show that neuroactive ligand-receptor interaction and the PPAR signaling pathway are highly associated with osteosarcoma.

In conclusion, the present study identified an eight-mRNA signature panel that can be considered a composite prognostic marker for survival risk stratification in osteosarcoma patients. This signature not only provides new insights into the molecular heterogeneity of osteosarcoma, but also has an independent prognostic value beyond conventional clinicopathological factors to predict the outcome of osteosarcoma. However, there are some limitations in our study that should be considered. The present research lacks effective validation in an independent cohort. We only analyzed TARGET data and the dependability of our findings has not been verified by *in vitro* or *in vivo* experiments. Further *in vitro* and *in vivo* approaches are needed to fully evaluate the role of the eight-mRNA signature in osteosarcoma.

## Author contributions

Zhaoming Ye and Bo Wu designed the study. Bo Wu, Zhan Wang, Nong Lin, Xiaobo Yan, Zhangchun Lv, Zhimin Ying performed the data collection and analysis. All authors participated in the writing of the manuscript. All the authors have read and approved the final version of this manuscript.

**Conceptualization:** Bo Wu, Zhaoming Ye, Zhangchun Lv, Zhimin Ying.

**Data curation:** Bo Wu, Zhan Wang, Nong Lin, Zhaoming Ye.

**Formal analysis:** Bo Wu, Zhan Wang, Nong Lin, Zhaoming Ye.

**Funding acquisition:** Zhan Wang, Zhaoming Ye.

**Investigation:** Bo Wu, Nong Lin, Xiaobo Yan, Zhaoming Ye.

**Methodology:** Bo Wu, Xiaobo Yan, Zhaoming Ye, Zhangchun Lv.

**Project administration:** Bo Wu, Zhan Wang, Nong Lin, Zhaoming Ye, Zhimin Ying.

**Resources:** Bo Wu, Zhan Wang, Nong Lin, Xiaobo Yan, Zhaoming Ye.

**Software:** Bo Wu, Zhaoming Ye.

**Supervision:** Bo Wu, Nong Lin, Zhaoming Ye, Zhangchun Lv.

**Validation:** Bo Wu, Zhan Wang, Zhaoming Ye.

**Visualization:** Bo Wu, Zhan Wang, Zhaoming Ye.

**Writing – original draft:** Bo Wu, Zhangchun Lv, Zhimin Ying.

**Writing – review & editing:** Bo Wu.

## References

[1] Duchman KR Gao Y Miller BJ. Prognostic factors for survival in patients with high-grade osteosarcoma using the Surveillance, Epidemiology, and End Results (SEER) Program database. Cancer Epidemiol 2015;39:593–9. 26002013 10.1016/j.canep.2015.05.001

[2] Sun W Wang W Lei J. Actin-like protein 6A is a novel prognostic indicator promoting invasion and metastasis in osteosarcoma. Oncol Rep 2017;37:2405–17. 28260090 10.3892/or.2017.5473

[3] Ding L Zhang G Hou Y. Elemene inhibits osteosarcoma growth by suppressing the renin-angiotensin system signaling pathway. Mol Med Rep 2018;17:1022–30. 29115494 10.3892/mmr.2017.7965

[4] Razzaghi H Quesnel-Crooks S Sherman R. Leading Causes of Cancer Mortality - Caribbean Region, 2003-2013. MMWR Morb Mortal Wkly Rep 2016;65:1395–400. 27977639 10.15585/mmwr.mm6549a3

[5] Kansara M Teng MW Smyth MJ. Translational biology of osteosarcoma. Nat Rev Cancer 2014;14:722–35. 25319867 10.1038/nrc3838

[6] Palmerini E Jones RL Marchesi E. Gemcitabine and docetaxel in relapsed and unresectable high-grade osteosarcoma and spindle cell sarcoma of bone. BMC Cancer 2016;16:280. 27098543 10.1186/s12885-016-2312-3 PMC4839113

[7] Anderson ME. Update on Survival in Osteosarcoma. Orthop Clin North Am 2016;47:283–92. 26614941 10.1016/j.ocl.2015.08.022

[8] Shao J Li Z Jiao G. Ganoderic acid A suppresses proliferation and invasion and induces apoptosis in human osteosarcoma cells. Nan Fang Yi Ke Da Xue Xue Bao 2015;35:619–24. 26018252

[9] Wang X Lai P Zhang Z. Targeted inhibition of mTORC2 prevents osteosarcoma cell migration and promotes apoptosis. Oncol Rep 2014;32:382–8. 24840134 10.3892/or.2014.3182

[10] Isakoff MS Bielack SS Meltzer P. Osteosarcoma: current treatment and a collaborative pathway to success. J Clin Oncol 2015;33:3029–35. 26304877 10.1200/JCO.2014.59.4895 PMC4979196

[11] Miller KDSRL Lin CCMAB Kramer JLRJH. Cancer treatment and survivorship statistics, 2016. CA Cancer J Clin 2016;66:271–89. 27253694 10.3322/caac.21349

[12] Misaghi A Goldin A Awad M. Osteosarcoma: a comprehensive review. SICOT J 2018;4:12. 29629690 10.1051/sicotj/2017028 PMC5890448

[13] Botter SM Neri D Fuchs B. Recent advances in osteosarcoma. Curr Opin Pharmacol 2014;16:15–23. 24632219 10.1016/j.coph.2014.02.002

[14] Zhu C Wei J Tian X. Prognostic role of PPAR-( and PTEN in the renal cell carcinoma. Int J Clin Exp Pathol 2015;8:12668–77. 26722456 PMC4680401

[15] Kluth M Runte F Barow P. Concurrent deletion of 16q23 and PTEN is an independent prognostic feature in prostate cancer. Int J Cancer 2015;137:2354–63. 26009879 10.1002/ijc.29613

[16] Mithal P Allott E Gerber L. PTEN loss in biopsy tissue predicts poor clinical outcomes in prostate cancer. Int J Urol 2014;21:1209–14. 25099119 10.1111/iju.12571

[17] Sleightholm RL Neilsen BK Li J. Emerging roles of the CXCL12/CXCR4 axis in pancreatic cancer progression and therapy. Pharmacol Ther 2017;179:158–70. 28549596 10.1016/j.pharmthera.2017.05.012 PMC13170075

[18] Şahpaz A Önal B Yeşilyurt A. BRAF(V600E) Mutation, RET/PTC1 and PAX8-PPAR Gamma Rearrangements in Follicular Epithelium Derived Thyroid Lesions - Institutional Experience and Literature Review. Balkan Med J 2015;32:156–66. 26167339 10.5152/balkanmedj.2015.15101 PMC4432695

[19] Cicenas J Tamosaitis L Kvederaviciute K. KRAS, NRAS and BRAF mutations in colorectal cancer and melanoma. Med Oncol 2017;34:26. 28074351 10.1007/s12032-016-0879-9

[20] Ahmed AR. HER2 expression is a strong independent predictor of nodal metastasis in breast cancer. J Egypt Natl Canc Inst 2016;28:219–27. 27756653 10.1016/j.jnci.2016.09.002

[21] Xiong M Wang L Yu HL. Ginkgetin exerts growth inhibitory and apoptotic effects on osteosarcoma cells through inhibition of STAT3 and activation of caspase-3/9. Oncol Rep 2016;35:1034–40. 26573608 10.3892/or.2015.4427

[22] Li C Guo D Tang B. Notch1 is associated with the multidrug resistance of hypoxic osteosarcoma by regulating MRP1 gene expression. Neoplasma 2016;63:734–42. 27468877 10.4149/neo_2016_510

[23] Yu L Fan Z Fang S. Cisplatin selects for stem-like cells in osteosarcoma by activating Notch signaling. Oncotarget 2016;7:33055–68. 27102300 10.18632/oncotarget.8849 PMC5078075

[24] Chen X Bahrami A Pappo A. Recurrent somatic structural variations contribute to tumorigenesis in pediatric osteosarcoma. Cell Rep 2014;7:104–12. 24703847 10.1016/j.celrep.2014.03.003 PMC4096827

[25] Sampson VB Gorlick R Kamara D. A review of targeted therapies evaluated by the pediatric preclinical testing program for osteosarcoma. Front Oncol 2013;3:132. 23755370 10.3389/fonc.2013.00132 PMC3668267

[26] Breyer J Wirtz RM Otto W. High PDL1 mRNA expression predicts better survival of stage pT1 non-muscle-invasive bladder cancer (NMIBC) patients. Cancer Immunol Immunother 2018;67:403–12. 29150702 10.1007/s00262-017-2093-9 PMC11028240

[27] Breyer J Otto W Wirtz RM. ERBB2 Expression as Potential Risk-Stratification for Early Cystectomy in Patients with pT1 Bladder Cancer and Concomitant Carcinoma in situ. Urol Int 2017;98:282–9. 27992871 10.1159/000453670

[28] Rinaldetti S Wirtz R Worst TS. FOXM1 predicts disease progression in non-muscle invasive bladder cancer. J Cancer Res Clin Oncol 2018;144:1701–9. 29959570 10.1007/s00432-018-2694-5 PMC6096766

[29] Uhercik M Sanders AJ Owen S. Clinical Significance of PD1 and PDL1 in Human Breast Cancer. Anticancer Res 2017;37:4249–54. 28739716 10.21873/anticanres.11817

[30] Xie H Zhu Y Zhang J. B4GALT1 expression predicts prognosis and adjuvant chemotherapy benefits in muscle-invasive bladder cancer patients. BMC Cancer 2018;18:590. 29793447 10.1186/s12885-018-4497-0 PMC5968541

[31] Brüggemann C Kirchberger MC Goldinger SM. Predictive value of PD-L1 based on mRNA level in the treatment of stage IV melanoma with ipilimumab. J Cancer Res Clin Oncol 2017;143:1977–84. 28616701 10.1007/s00432-017-2450-2 PMC11819317

[32] Li Q Li F Che J. Expression of B7 Homolog 1 (B7H1) Is Associated with Clinicopathologic Features in Urothelial Bladder Cancer. Med Sci Monit 2018;24:7303–8. 30315148 10.12659/MSM.910956 PMC6196594

[33] Jin PY Lu HJ Tang Y. The effect of DNA-PKcs gene silencing on proliferation, migration, invasion and apoptosis, and in vivo tumorigenicity of human osteosarcoma MG-63 cells. Biomed Pharmacother 2017;96:1324–34. 29203385 10.1016/j.biopha.2017.11.079

[34] Li X Xu R Liu H. CUL4A expression in pediatric osteosarcoma tissues and its effect on cell growth in osteosarcoma cells. Tumour Biol 2016;37:8139–44. 26715273 10.1007/s13277-015-4715-1

[35] Zhang B Li YL Zhao JL. Hypoxia-inducible factor-1 promotes cancer progression through activating AKT/Cyclin D1 signaling pathway in osteosarcoma. Biomed Pharmacother 2018;105:01–9. 10.1016/j.biopha.2018.03.165 29807229

[36] Shen A Chen Y Liu L. EBF1-mediated upregulation of ribosome assembly factor PNO1 contributes to cancer progression by negatively regulating the p53 signaling pathway. Cancer Res 2019;79:2257–70. 30862720 10.1158/0008-5472.CAN-18-3238

[37] Peng T Li Z Li D. MACC1 promotes angiogenesis in cholangiocarcinoma by upregulating VEGFA. Onco Targets Ther 2019;12:1893–903. 30881041 10.2147/OTT.S197319 PMC6415730

[38] Eisen MB Spellman PT Brown PO. Cluster analysis and display of genome-wide expression patterns. Proc Natl Acad Sci U S A 1998;95:14863–8. 9843981 10.1073/pnas.95.25.14863 PMC24541

[39] Zan XY Li L. Construction of lncRNA-mediated ceRNA network to reveal clinically relevant lncRNA biomarkers in glioblastomas. Oncol Lett 2019;17:4369–74. 30944630 10.3892/ol.2019.10114 PMC6444437

[40] Urrego D Tomczak AP Zahed F. Potassium channels in cell cycle and cell proliferation. Philos Trans R Soc Lond B Biol Sci 2014;369:20130094. 24493742 10.1098/rstb.2013.0094 PMC3917348

[41] Andrejeva D Kugler JM Nguyen HT. Metabolic control of PPAR activity by aldehyde dehydrogenase regulates invasive cell behavior and predicts survival in hepatocellular and renal clear cell carcinoma. BMC Cancer 2018;18:1180. 30486822 10.1186/s12885-018-5061-7 PMC6264057

[42] Zhang N Chu ES Zhang J. Peroxisome proliferator activated receptor alpha inhibits hepatocarcinogenesis through mediating NF-(B signaling pathway. Oncotarget 2014;5:8330–40. 25327562 10.18632/oncotarget.2212 PMC4226686

[43] Xu J Li D Cai Z. An integrative analysis of DNA methylation in osteosarcoma. J Bone Oncol 2017;9:34–40. 29234590 10.1016/j.jbo.2017.05.001 PMC5715438

